# A Conserved Helicase Processivity Factor Is Needed for Conjugation and Replication of an Integrative and Conjugative Element

**DOI:** 10.1371/journal.pgen.1003198

**Published:** 2013-01-10

**Authors:** Jacob Thomas, Catherine A. Lee, Alan D. Grossman

**Affiliations:** Department of Biology, Massachusetts Institute of Technology, Cambridge, Massachusetts, United States of America; University of Geneva Medical School, Switzerland

## Abstract

Integrative and conjugative elements (ICEs) are agents of horizontal gene transfer and have major roles in evolution and acquisition of new traits, including antibiotic resistances. ICEs are found integrated in a host chromosome and can excise and transfer to recipient bacteria via conjugation. Conjugation involves nicking of the ICE origin of transfer (*oriT*) by the ICE–encoded relaxase and transfer of the nicked single strand of ICE DNA. For ICE*Bs1* of *Bacillus subtilis*, nicking of *oriT* by the ICE*Bs1* relaxase NicK also initiates rolling circle replication. This autonomous replication of ICE*Bs1* is critical for stability of the excised element in growing cells. We found a conserved and previously uncharacterized ICE gene that is required for conjugation and replication of ICE*Bs1*. Our results indicate that this gene, *helP* (formerly *ydcP*), encodes a helicase processivity factor that enables the host-encoded helicase PcrA to unwind the double-stranded ICE*Bs1* DNA. HelP was required for both conjugation and replication of ICE*Bs1*, and HelP and NicK were the only ICE*Bs1* proteins needed for replication from ICE*Bs1 oriT*. Using chromatin immunoprecipitation, we measured association of HelP, NicK, PcrA, and the host-encoded single-strand DNA binding protein Ssb with ICE*Bs1*. We found that NicK was required for association of HelP and PcrA with ICE*Bs1* DNA. HelP was required for association of PcrA and Ssb with ICE*Bs1* regions distal, but not proximal, to *oriT*, indicating that PcrA needs HelP to progress beyond nicked *oriT* and unwind ICE*Bs1*. In vitro, HelP directly stimulated the helicase activity of the PcrA homologue UvrD. Our findings demonstrate that HelP is a helicase processivity factor needed for efficient unwinding of ICE*Bs1* for conjugation and replication. Homologues of HelP and PcrA-type helicases are encoded on many known and putative ICEs. We propose that these factors are essential for ICE conjugation, replication, and genetic stability.

## Author Summary

Integrative and conjugative elements (ICEs) are mobile DNA elements that transfer genetic material between bacteria, driving bacterial evolution and the acquisition of new traits, including the spread of antibiotic resistances. ICEs typically reside integrated in a bacterial chromosome and are passively propagated along with the host genome. Under some conditions, an ICE can excise from the chromosome to form a circle and, if appropriate recipient bacteria are present, can transfer from donor to recipient. It has recently been recognized that some, and perhaps many, ICEs undergo autonomous replication after excision from the host chromosome and that replication is important for stability and propagation of these ICEs in growing cells. Using ICE*Bs1*, an ICE from *Bacillus subtilis*, we found a conserved and previously uncharacterized ICE gene that is required for conjugation and replication. We found that this gene, *helP*, encodes a helicase processivity factor that associates with ICE*Bs1* DNA and enables the host-encoded helicase PcrA to unwind the double-stranded ICE*Bs1* DNA, making a template for both conjugation and DNA replication. Homologues of *helP* are found in many ICEs, indicating that this mechanism of unwinding is likely conserved among these elements.

## Introduction

Integrative and conjugative elements (ICEs), also known as conjugative transposons, are mobile genetic elements that play a significant role in bacterial evolution and the acquisition of new traits [1]. They contribute significantly to the spread of antibiotic resistances in pathogenic bacteria. ICEs or putative ICEs are found in all major bacterial clades [2]. They reside integrated in a host genome and are propagated along with the host chromosome. Under certain conditions, an ICE can excise from the chromosome, form a double-stranded DNA (dsDNA) circle, and transfer to a recipient. Like conjugative plasmids, ICEs encode a multi-component mating pore complex that mediates their transfer from donors to recipients. Most ICEs are thought to transfer linear ssDNA. Transfer is through a type IV secretion system in Gram negative bacteria [3], [4], or its counterpart in Gram positive bacteria [5]. ICEs that transfer ssDNA were generally thought to lack the ability to undergo autonomous replication. However, recent work [6]–[8] and findings presented here indicate that autonomous replication is a property of many ICEs and that the mechanisms are conserved.

ICE*Bs1* is approximately 20 kb and normally found integrated in the tRNA gene *trnS-leu2* of *Bacillus subtilis* [9], [10]. ICE*Bs1* gene expression and excision can be induced in >90% of cells in a population by overproduction of the activator and cell signaling regulator RapI [9]. Following induction, ICE*Bs1* undergoes autonomous plasmid-like rolling circle replication [6]. Replication of ICE*Bs1* is needed for stability of the element after excision [6].

ICE*Bs1* replication and conjugation both begin with nicking of the ICE*Bs1* origin of transfer, *oriT*, by the ICE*Bs1*-encoded relaxase NicK. The nicked DNA is then unwound by the host-encoded helicase PcrA, rather than the replicative helicase (*B. subtilis* DnaC) [6]. During rolling-circle plasmid replication, the free 3′-OH of the nicked strand acts as a primer for replication by the host DNA polymerase, followed by recircularization of and complementary strand synthesis from the unwound single strand. By analogy to other conjugative systems, the single strand of ICE*Bs1* DNA covalently attached to the relaxase can also be targeted to the mating machinery by the putative coupling protein ConQ and transferred into recipient cells. Although unwinding of ICE*Bs1* DNA by the PcrA helicase is essential for both replication and conjugation of ICE*Bs1*, replication of the element in donor cells is not required for its transfer to recipients [6].

PcrA-type helicases (PcrA from Gram positive bacteria and UvrD and Rep from *E. coli*) are required for rolling circle replication of many different plasmids and phages. The PcrA-type proteins are efficient and processive DNA translocases, but typically have poor helicase activity. For many of the characterized phages and plasmids, the element-encoded relaxase that is needed for initiation of replication interacts with the PcrA-type helicase to stimulate DNA unwinding {reviewed in [11], [12], [13]}.

Unlike these other relaxases, we found that the ICE*Bs1* relaxase NicK was not sufficient for ICE*Bs1* replication. In addition to *nicK*, a second ICE*Bs1* gene (*helP*, previously *ydcP*) was necessary for replication from ICE*Bs1 oriT*. Expression of both *nicK* and *helP* in *B. subtilis* was sufficient to support replication from *oriT*. *helP* encodes a protein of previously unknown function and is conserved in many ICEs. We found that HelP is required for both mating and replication of ICE*Bs1*, and that it stimulates the function of the helicase PcrA. We also found that the *E. coli* helicase UvrD (a homologue of PcrA) can substitute for PcrA in *B. subtilis*, to support both cell viability and ICE*Bs1* conjugation and replication. Based on in vivo and in vitro analyses, HelP is a helicase processivity factor that is needed for efficient unwinding of ICE*Bs1*.

*helP* homologues are found in many ICEs, often in a module with genes encoding the relaxase and the putative coupling protein, indicating that these ICEs may also be capable of autonomous replication. PcrA homologues are also found on many extrachromosomal elements, either separately or as a helicase domain attached to the relaxase, indicating that these elements all share a need for DNA unwinding that is met in different ways.

## Results

### Two ICE*Bs1* genes, *nicK* and *helP*, are sufficient for autonomous replication from the ICE*Bs1 oriT* in *B. subtilis*

Studies of several rolling-circle plasmids have shown that only one plasmid gene, encoding the plasmid relaxase, is required for replication from the cognate origin of replication {reviewed in [12]}. In contrast, we found that the ICE*Bs1* relaxase NicK was not sufficient for autonomous replication from the ICE*Bs1* origin of replication *oriT*. Instead, a second ICE*Bs1* gene, *helP*, was also needed.

To determine which ICE*Bs1* genes are needed for replication from *oriT*, we constructed a series of plasmids that carry the ICE*Bs1 oriT* along with various candidate ICE*Bs1* genes. We then tested each plasmid for its ability to replicate in *B. subtilis*. The parent plasmid, pUS19 [14], carries a pUC-derived origin of replication that is not functional in *B. subtilis*, but is functional in *E. coli*, allowing purification of each test plasmid from *E. coli*. pUS19 also carries *spcE*, which allowed us to transform each plasmid into *B. subtilis* and select for spectinomycin-resistant transformants that stably acquired the plasmid. Transcription of the ICE*Bs1* genes was driven from the ICE*Bs1* promoter Pxis that was cloned onto the plasmid. Pxis is derepressed in cells lacking ICE*Bs1*.

After analyzing various plasmids containing different combinations of candidate ICE*Bs1* genes (data not shown), we found that *helP* and *nicK* were sufficient to support replication from ICE*Bs1 oriT* (Table 1). A plasmid containing *oriT*, *nicK*, and *helP* (pCAL1255) was capable of transforming a *B. subtilis* strain lacking ICE*Bs1* (Table 1, pCAL1255). The plasmid copy number was between 25–30 (Table 1) as indicated by the amount of *spcE* DNA (plasmid) relative to *ydbT*, a chromosomal gene adjacent to the ICE*Bs1* attachment site *attB*.

**Table 1:** NicK and HelP are the only ICE–encoded factors required for autonomous replication.

	ICE*Bs1* genes in^1^		Relative copy number of plasmid^2^
Line	Plasmid	Chromosome	
1	*helP, nicK*	None	27±3.5
2	*helP*	None	no replication
3	*helP*	*nicK*	4.1±0.2
4	*nicK*	None	no replication
5	*nicK*	*helP*	76±8.3

1 Plasmids containing *spcE* and ICE*Bs1 oriT* also contained the indicated genes, *helP* and/or *nicK*, under control of ICE*Bs1* Pxis. *nicK* or *helP* was integrated in the chromosome as indicated.

2 Plasmids were isolated from *E. coli* and transformed into *B. subtilis* cells that contained no ICE*Bs1* (ICE*Bs1* ^0^; strain JMA222 or derivatives). In strains with transformants, the relative copy number of the plasmid was determined by measuring the relative amount of *spcE* DNA. Transformants were obtained if the plasmids were capable of replication, only when both *nicK* and *helP* were present. To ensure that the plasmids were transformable, constructs were also transformed into IRN342 (containing ICE*Bs1*) where gene expression is repressed by ImmR and the plasmids are able to integrate into the host chromosome. All plasmids were capable of transforming this recipient, and all had a relative copy number of one. Data presented are averages from at least 3 independent experiments ± standard deviation.

Replication of pCAL1255 (*oriT*, Pxis-*helP*-*nicK*) in *B. subtilis* was dependent on expression of *helP* and *nicK* from Pxis. We repressed expression from Pxis by transforming pCAL1255 into *B. subtilis* carrying an intact integrated ICE*Bs1*, which expresses the ICE*Bs1* repressor ImmR. We were still able to obtain spectinomycin-resistant transformants of pCAL1255 in the ICE*Bs1*-containing cells. However, in these transformants, the plasmid copy number was one, indicating that the plasmid was likely integrated into the chromosomal ICE*Bs1* by homologous recombination.

We found that autonomous plasmid replication from *oriT* was dependent on both *helP* and *nicK*. We did not obtain any spectinomycin-resistant transformants in *B. subtilis* cells lacking ICE*Bs1* from plasmids containing *nicK* without *helP* (Table 1, pJT151) or *helP* without *nicK* (Table 1, pCAL1260). However, we were able to transform these plasmids into strains that expressed the missing gene from a chromosomal locus, showing that the replication defects could be complemented. pJT151 (*oriT*, Pxis-*nicK*) was able to replicate and had a copy number of 70–80 in a strain expressing *helP* from the chromosome. pCAL1260 (*oriT*, Pxis-*helP*) was able to replicate and had a copy number of approximately 4 in a strain expressing *nicK* from the chromosome (Table 1). The different copy numbers of pCAL1255 (*oriT*, Pxis-*helP-nicK*) and the plasmids missing *helP* or *nicK* but complemented from a chromosomal copy of the gene may be due to different expression levels of HelP and NicK from the plasmid versus the chromosome, or the effects of the different plasmid sequences on replication efficiency. The low copy number when *nicK* was expressed from the chromosome is also consistent with the notion that the relaxase functions preferentially in cis [15].

Based on these results, we conclude that, unlike many rolling circle plasmid replicons, the ICE*Bs1* relaxase NicK is not the only element-encoded protein needed for autonomous replication. ICE*Bs1*-encoded NicK and HelP are both needed to support replication from *oriT* in the absence of other ICE*Bs1* products.

### *helP* is required for autonomous replication of ICE*Bs1*

**Figure 1:**
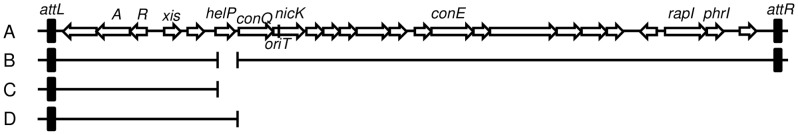
Map of ICE*Bs1* and derivatives. A–B. Schematic of ICE*Bs1* (A) and the *helP* mutant (B) integrated at the normal attachment site, *attB*. The antibiotic resistance marker (*kan*) that is inserted in *rapI-phrI* is not shown. A. Genetic map of ICE*Bs1*. The linear integrated form of ICE*Bs1* is shown. Open arrows indicate open reading frames and the direction of transcription. The black rectangles at the ends of ICE*Bs1* represent the 60 bp direct repeats that contain the site-specific recombination sites in the left and right attachment sites, *attL* and *attR*. Some gene names are indicated. The genes encoding the ICE*Bs1* repressor *immR* and anti-repressor *immA* are indicated by *R* and *A*, respectively. The origin of transfer (*oriT*) is indicated by a black line in the 5′ end of *nicK*. *oriT* is needed for ICE*Bs1* transfer [15] and replication [6]. Primers used in ChIP-PCR experiments hybridize to *nicK* (*oriT*), and *conE*. B. Schematic of the Δ*helP155* mutation in ICE*Bs1*. Thin horizontal lines represent the regions of ICE*Bs1* that are present in the mutant. The gap in the line represents the in-frame markerless deletion of *helP*. C–D. Diagram of the truncated ICE*Bs1* derivatives that were used to test complementation of Δ*helP* donors. Both constructs are integrated at *thrC* and contain ICE*Bs1* genes represented by the horizontal lines, from *attL* to *ydcO* (C) or *helP* (D). Both derivatives contain *cat* (chloramphenicol resistance) in place of the part of ICE*Bs1* that is deleted, and neither can excise from the chromosome due to the absence of *attR*.

We found that *helP* is required for replication of ICE*Bs1*. We constructed an in-frame markerless deletion of *helP* (Δ*helP*) that removed its entire coding sequence from ICE*Bs1* (Figure 1B). After inducing ICE*Bs1* gene expression, we measured ICE*Bs1* copy number by quantitative real time PCR (qPCR). The copy number of ICE*Bs1* relative to the chromosome is expressed as the relative amount of DNA from ICE*Bs1 oriT* compared to *ydbT*, as described previously [6]. Consistent with previous findings, the relative copy number of ICE*Bs1 oriT* was 3–4 per cell one hour after induction of ICE*Bs1* gene expression (Table 2, line 1). Under similar conditions, the copy number of the ICE*Bs1* Δ*helP* mutant was approximately 0.5 (Table 2, line 2). A copy number less than one is consistent with previous findings that replication-defective ICE*Bs1* is progressively lost from a population of dividing cells [6].

**Table 2:** HelP is required for ICE*Bs1* transfer and replication.

ICE*Bs1* Donor	Mating Efficiency (%)	Relative copy number of *oriT*
wild type (JMA168)	3.67±1.88	3.45±0.49
Δ*helP* (JT155)	<0.000009	0.49±0.14
Δ*helP thrC*::*helP*Δ (JT334)	<0.000009	0.58±0.15
Δ*helP thrC*::*helP*+ (JT335)	0.11±0.05	2.36±0.15

Mating efficiencies and copy numbers (averages from at least 3 independent experiments ± standard deviation) were measured one hour after induction of ICE*Bs1* gene expression by overproduction of RapI from *amyE*::{Pspank(hy)-*rapI spc*}. Copy number of *oriT* is expressed relative to a control region *ydbT* adjacent to ICE*Bs1.* Wild type and all derivates of ICE*Bs1* contained Δ(*rapI*-*phrI*)::*kan*. The ICE*Bs1* constructs at *thrC* contained the left end of ICE*Bs1* and extend to immediately before *helP* (*thrC*::*helpΔ*; Figure 1C) or to the end of *helP* (*thrC*::*helP* ^+^; Figure 1D).

### *helP* is required for ICE*Bs1* conjugation and functions primarily in donor cells

In order to determine the stage at which HelP functions in ICE*Bs1* replication, we tested whether *helP* is required for transfer of ICE*Bs1* into a recipient. Although ICE*Bs1* replication is not required for mating, the initial steps of nicking and unwinding of ICE*Bs1* DNA are common to both mating and replication [6], [15]. Wild type ICE*Bs1* had a mating efficiency of 3.7% (3.7 transconjugants per 100 donors) (Table 2, line 1), similar to previous results [9], [16]–[18]. In contrast, transfer of the ICE*Bs1* Δ*helP* mutant was undetectable (Table 2, line 2). Expression of *helP* from a truncated ICE*Bs1* integrated into the chromosome at *thrC* (strain JT335; Figure 1) largely restored conjugation, indicating that the primary mating defect was due to loss of *helP* and not due to polarity on downstream genes (Table 2 line 3).

Although HelP primarily functions in the donor, it also appears to play a role in the recipient. We found that expression of *helP* in the recipient from the IPTG-inducible promoter Pspank(hy) increased the mating efficiency to 1.4%, from the 0.11% efficiency when *helP* was provided only in the donor. There was no detectable transfer of the ICE*Bs1* Δ*helP* mutant unless *helP* was also expressed in the donor, and no increase in mating efficiency of wild type ICE*Bs1* when *helP* was also expressed in the recipient. We suspect that HelP is required for autonomous replication and increases the stability of ICE*Bs1* in recipient cells prior to its integration into the recipient chromosome.

Since HelP was required for both conjugation and replication of ICE*Bs1*, we expected it to be involved in nicking or unwinding of ICE*Bs1* DNA. There was still nicking of the proper site in *oriT* in the absence of *helP* (data not shown), consistent with previous findings that NicK is the only ICE*Bs1*-encoded protein needed for nicking of *oriT* [15]. Therefore we suspected that HelP was involved in the unwinding of ICE*Bs1* DNA by the host-encoded helicase PcrA, a DNA translocase that has very limited processivity as a helicase [19]. The solution structure of a HelP homologue, SAG0934 from *Streptococcus agalactiae* [20], which is identical to Orf22 of Tn*916* from *Enterococcus faecalis*, indicates that HelP contains an oligonucleotide/oligosaccharide binding fold (OB-fold) that is present in many ssDNA binding proteins [21], consistent with a possible role in binding ICE*Bs1* ssDNA. Although HelP homologues and some Ssb proteins share the OB-fold, there appear to be no other significant sequence similarities between these proteins.

### HelP associates with ICE*Bs1 oriT*

**Figure 2:**
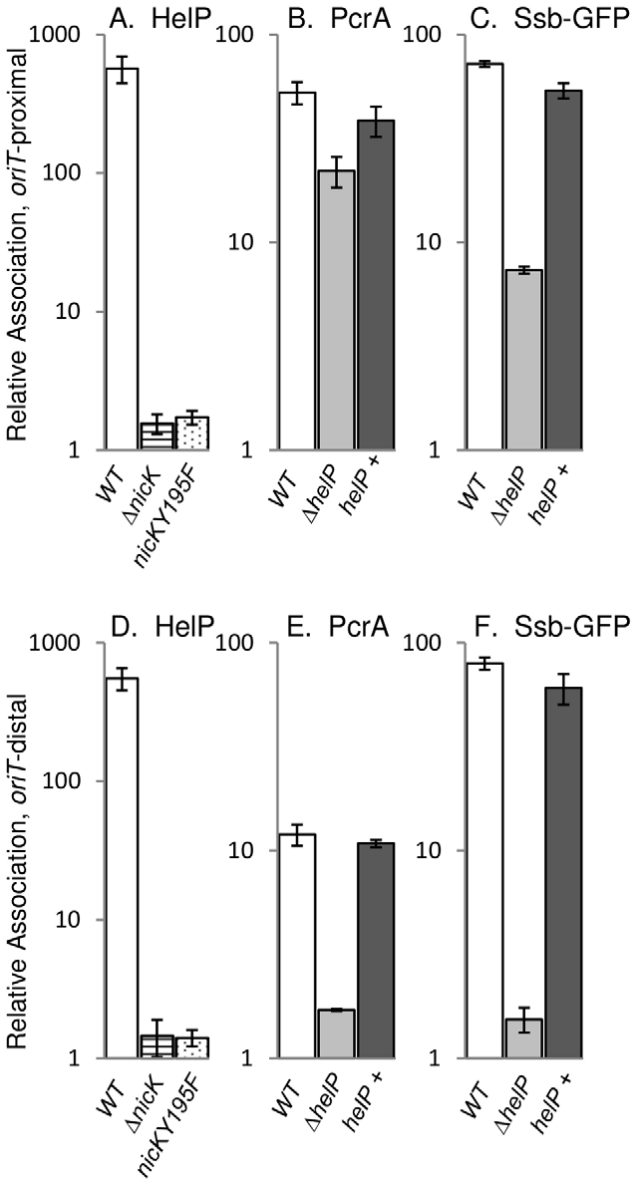
HelP, PcrA, and Ssb-GFP association with ICE*Bs1* DNA. ICE*Bs1* gene expression was induced by overproduction of RapI from *amyE*::{Pspank(hy)-*rapI spc*} for one hour followed by DNA-protein crosslinking with formaldehyde. Association of HelP (A, D), PcrA (B, E), and Ssb-GFP (C, F) with DNA sequences near *oriT* (A–C) and *conE* (D–F) were measured by immunoprecipitation of lysates with polyclonal anti-HelP, anti-PcrA and anti-GFP antibodies respectively followed by qPCR (ChIP-PCR), relative to *ydbT*, adjacent to ICE*Bs1*. When Ssb-GFP association was measured, strains contained the *lacA*::{PrpsF-*ssb-GFPm tet*} allele expressing GFP-tagged Ssb [6]. Strains included: wild type (JMA168 for HelP and PcrA; MMB834 for Ssb-GFP), white bars; *ΔnicK* (CAL306), horizontally striped bars; *nicKY195F* (JT340), dotted bars; Δ*helP* (JT155 for HelP and PcrA; JT252 for Ssb-GFP), light grey bars; and Δ*helP*, complemented with *helP+* (JT335 for HelP and PcrA; JT398 for Ssb-GFP), dark grey bars. The c-Myc tag in the *nicKY195F* allele did not affect HelP association with *conE* which was unchanged in the NicK-Myc (JT308) control strain (421±96) compared to wild type (551±100) in three independent experiments. Error bars represent standard deviation.

Using crosslinking and immunoprecipitation (ChIP), we found that HelP was associated with ICE*Bs1 oriT in vivo* (Figure 2). Following induction of ICE*Bs1* gene expression, protein and DNA were crosslinked with formaldehyde and HelP was immunoprecipitated with polyclonal anti-HelP antibodies. Preliminary experiments with DNA microarrays (ChIP-chip) indicated that HelP was strongly associated with the excised and replicating ICE*Bs1* DNA and that there was little or no specific association with the chromosome (data not shown). We then measured association of HelP with ICE*Bs1* DNA following ChIP using quantitative real time PCR (ChIP-PCR) with primers specific to ICE*Bs1 oriT* and normalized to the amount of DNA from a chromosomal region (Materials and Methods). *oriT* DNA was enriched >500-fold in the anti-HelP immunoprecipitates (Figure 2A) indicating association of HelP with ICE*Bs1 oriT in vivo*. The immunoprecipitation was specific to HelP as ICE*Bs1 oriT* was not significantly enriched in a strain deleted for *helP* (2.4-fold relative association in *ΔhelP* mutant compared to >500-fold in wild type).

### Association of HelP with ICE*Bs1 oriT* depends on the ICE*Bs1* relaxase NicK

We found that in vivo, association of HelP with ICE*Bs1 oriT* required the activity of the relaxase NicK. In a strain containing a *nicK* deletion, and a functional *oriT* [15], association of HelP with ICE*Bs1* was abolished (Figure 2A). To test if NicK nicking activity was required for association of HelP at *oriT*, we made a mutation in the catalytic site of NicK that changes the conserved tyrosine at position 195 to phenylalanine (*nicKY195F*). We also incorporated a C-terminal 3× c-Myc tag onto the wild type and Y195F mutant NicK proteins to use in ChIP experiments with monoclonal antibodies to c-Myc. Both NicKY195F-Myc NicK-Myc were associated with ICE*Bs1 oriT* as determined by ChIP-PCR experiments (40±2-fold enrichment and 44±4-fold enrichment, respectively). As expected, there was no detectable nicking of *oriT* by NicKY195F-Myc (<0.05%±0.03% nicked *oriT*) whereas there was normal nicking of *oriT* by NicK-Myc (32%±3.7% nicked *oriT*), as determined by primer extension assays [15]. The NicK-Myc was also functional in conjugation (Materials and Methods).

We measured the association of HelP with ICE*Bs1 oriT* in vivo in strains expressing either NicK-Myc or NicKY195F-Myc. As expected, HelP was associated with ICE*Bs1 oriT* in the strain expressing functional NicK-Myc (data not shown). In contrast, HelP was not detectably associated with ICE*Bs1 oriT* in the strain expressing NicKY195F-Myc (Figure 2A). These results indicate that the presence of NicK at *oriT* is not sufficient and that nicking of *oriT* is required for recruitment of HelP to *oriT*. We also found that HelP was associated with the rolling circle replicating plasmid pBS42 (data not shown), consistent with the notion that HelP does not require specific interaction with NicK.

### HelP is not required for recruitment of the helicase PcrA to ICE*Bs1 oriT* in vivo

The host-encoded helicase PcrA is required for both conjugation and replication of ICE*Bs1* [6]. Since HelP is also required for conjugation and replication of ICE*Bs1*, we tested for effects of HelP on PcrA. We measured association of PcrA with ICE*Bs1 oriT* by ChIP-PCR using polyclonal anti-PcrA antibodies and primers specific to *oriT* as described above. PcrA association with *oriT* was not significantly affected in a Δ*helP* mutant, compared to wild type (Figure 2B). We conclude that association of PcrA with ICE*Bs1 oriT* is not dependent on HelP.

In contrast, we found that association of PcrA with ICE*Bs1 oriT* appeared to be largely, but not entirely, dependent on nicking of *oriT* by NicK. Enrichment of *oriT* in the PcrA immunoprecipitates was reduced but not abolished in the *nicKY195F-myc* mutant (approximately 6-fold enrichment in *nicKY195F-myc* vs 65–70-fold enrichment with *nicK-myc*). Association of PcrA with ICE*Bs1* DNA in the *nicK* catalytic site mutant indicates that there might be interactions between ICE*Bs1* NicK and the host PcrA, as there are with other replicative rolling circle relaxases and PcrA [22], [23]. The immunoprecipitation was specific for PcrA; in a *pcrA recF* double mutant [6], ICE*Bs1* sequences were not significantly enriched (approximately 1.1-fold relative enrichment of *oriT* compared to 12.5-fold enrichment in the *recF* parent). (Loss of *recF* suppresses the lethality caused by loss of *pcrA* [24], [25]).

### HelP is required for progression of the helicase PcrA through ICE*Bs1* DNA in vivo

Unwinding and replication of ICE*Bs1* DNA by PcrA proceeds unidirectionally from the nicked *oriT* [6]. Replication of ICE*Bs1* is also accompanied by association of the host-encoded single stranded DNA binding protein (Ssb) to the ICE*Bs1* DNA [6]. To examine the location and role of HelP during unwinding and replication of ICE*Bs1* DNA from the nicked *oriT*, we compared association of HelP, PcrA and Ssb with an *oriT*-proximal (Figure 2A–2C) to an *oriT*-distal region (Figure 2D–2E).

We found that although HelP was not needed for the initial recruitment of PcrA to *oriT* (Figure 2B), it was required for the association of PcrA with *oriT*-distal regions (Figure 2E). Association of HelP and PcrA with the *oriT*-distal (*conE*) region of ICE*Bs1* DNA was readily detectable in wild type (*helP* ^+^) cells (Figure 2D, 2E). In contrast, association of PcrA with ICE*Bs1* DNA in the Δ*helP* mutant was greatly reduced in this region (Figure 2E). This reduction was due to loss of *helP* and not an unexpected secondary effect due to polarity or alterations in the DNA because association was restored when *helP* was expressed in trans from an ectopic locus (Figure 2E). Together, our results indicate that HelP is not needed for the initial association of PcrA with ICE*Bs1 oriT*, but that HelP is needed for PcrA to become associated with distal regions, perhaps by affecting helicase processivity.

We monitored association of Ssb with ICE*Bs1* DNA (indicative of unwound ssDNA) using an Ssb-GFP fusion and ChIP-PCR with anti-GFP antibodies, essentially as described previously [6]. We found that Ssb-GFP was associated with both the *oriT*-proximal and *oriT*-distal (*conE*) region of ICE*Bs1* (Figure 2C, 2E). In contrast, there was little or no association of Ssb-GFP with the *oriT*-distal region in the *helP* mutant (Figure 2E), and association was reduced, but still appreciable, in the *oriT* region (Figure 2C). Association of Ssb with ICE*Bs1* DNA was restored by expression of *helP* from an ectopic locus (Figure 2C, 2E), indicating that the defect in the *helP* mutant was due to loss of *helP* and not some unexpected effect. Based on these results, we conclude that HelP is required for both PcrA and Ssb to associate with *oriT*-distal sequences in ICE*Bs1*, and that HelP is likely needed for the processive unwinding of ICE*Bs1* DNA. This function would be sufficient to explain the requirement for *helP* in both conjugation and replication of ICE*Bs1*.

### HelP stimulates helicase activity of the PcrA homologue UvrD in vitro

We wished to test directly the ability of HelP to facilitate unwinding of duplex DNA by PcrA. Our many different preparations of *B. subtilis* PcrA were of low concentration and rapidly lost helicase activity. Since most structural and biochemical analyses of PcrA have been done with a homologue from another organism {reviewed in [26]}, we decided to test for effects of HelP on UvrD from *E. coli*. UvrD is a well-studied homologue of PcrA and has 41% identity with *B. subtilis* PcrA. Like PcrA, UvrD is required for replication of several rolling circle replicating plasmids [27]. We found that expression of *E. coli uvrD* from the IPTG-inducible promoter Pspank (Pspank-*uvrD*) in *B. subtilis* suppressed the lethality caused by loss of *pcrA*. This suppression occurred in both the absence and the presence of IPTG, indicating that, in the absence of induction, expression from Pspank was leaky and sufficient levels of UvrD were produced. In addition, UvrD was able to support replication and conjugation of ICE*Bs1* nearly as well as PcrA (Table 3). In cells missing *pcrA* but expressing *uvrD*, the mating efficiency was approximately 1% (transconjugants per donor) and ICE*Bs1* was capable of replication and had a copy number of 2–3 (Table 3 line 3). Based on these results, we conclude that *E. coli uvrD* can replace *pcrA* in *B. subtilis* and provides the functions of PcrA needed for cell viability and those needed for ICE*Bs1* conjugation and replication.

**Table 3:** UvrD permits ICE*Bs1* replication in a *pcrA*-defective strain.

ICE*Bs1* Donor (strain number)	Percent mating	Relative copy number of *oriT*
wild type (CAL1772)	1.71±0.17	10.09±0.39
*uvrD*+ (CAL1773)	5.38±1.97	10.11±0.67
Δ*pcrA uvrD+* (CAL1686)	1.18±0.29	2.76±0.01

Induction of ICE*Bs1* gene expression was carried out by overproduction of RapI from *amyE*::{Pxyl-*rapI spc*} for two hours followed by mating as described in Materials and Methods. Data presented are averages from two experiments ± standard deviation. Wild type and all derivates of ICE*Bs1* contained Δ(*rapI*-*phrI*)::*kan*. All strains were also *thrC* mutants, with either *thrC*::*cat* or *E. coli uvrD* cloned and integrated into *thrC* (*thrC*::Pspank-*uvrD mls*).

**Figure 3:**
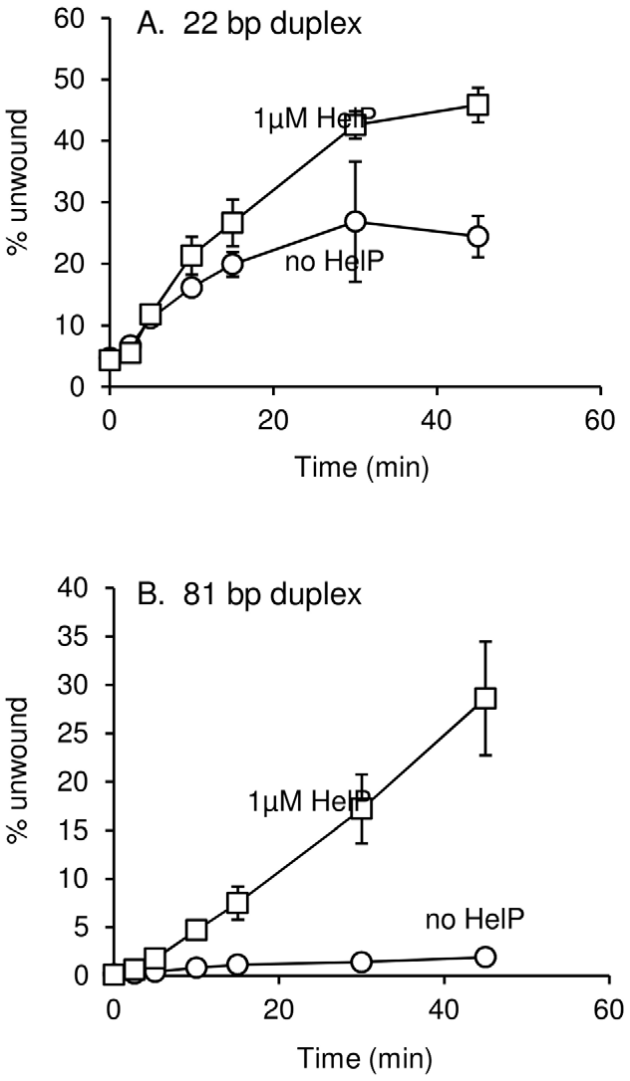
DNA unwinding by UvrD is stimulated by HelP. Substrates for unwinding were M13mp18 ssDNA annealed to either a 22 (A) or 81 (B) complementary fluorophore-labeled oligonucleotide. Data presented are the averages of three independent experiments ± standard deviation. Substrate unwinding was undetectable in the presence of HelP alone (<0.5% substrate unwound after 45 minutes) for both substrates. These controls were done with only the 45 minute time point.

We found that HelP stimulated the ability of UvrD to unwind a partial duplex DNA substrate in vitro. We purified hexahistidine-tagged UvrD (his-UvrD) and HelP (his-HelP) and used two different partial duplex DNA substrates to monitor unwinding (Materials and Methods). The substrates had either a small (22 nucleotides) or large (81 nucleotides) fluorophore-labeled oligonucleotide hybridized to single-stranded M13mp8 DNA. Helicase activity was measured by release of the labeled oligonucleotide, detected by gel electrophoresis and fluorometry. HelP alone had no effect on the partial duplex substrate. In both cases, <0.5% of the substrate duplex was unwound after 45 minutes. UvrD alone was able to unwind the 22 bp duplex: approximately 20% of the substrate was unwound after 45 minutes (Figure 3A). The addition of HelP increased the amount of substrate that was unwound to approximately 40% (Figure 3A). In contrast, there was little or no release of the large (81-mer) oligonucleotide from the partial duplex by UvrD alone (<2% unwound after 45 minutes). In the presence of HelP and UvrD, >25% of the 81 bp duplex substrate was unwound by 45 minutes (Figure 3B). Based on the increase in unwinding and the difference in stimulation between the 22 bp and 81 bp duplex substrates, we conclude that HelP stimulates the ability of UvrD, and likely PcrA, to processively unwind duplex DNA. In addition, since there is no relaxase in this assay, relaxase, and specifically NicK, is not required, at least in vitro, for the function of HelP.

### Conservation of *helP* in many ICEs

**Figure 4:**
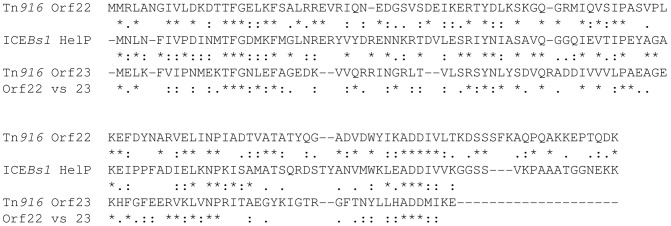
Comparison of HelP to Orf22 and Orf23 of Tn*916*. HelP (middle sequence), Orf22 of Tn*916* (top sequence) and Orf23 of Tn*916* (bottom sequence) were aligned using Clustal X. Markings above and below the HelP sequence indicate which amino acids are identical (asterisk), highly similar (colon) or weakly similar (period) to Tn*916* Orf22 (marks above) and Tn*916* Orf23 (marks below). Markings for the pairwise comparison of Orf22 to Orf23 are shown below the Tn*916* Orf23 sequence. Needleman-Wunsch alignment scores indicate that HelP is more similar to Orf22 and Orf23 (N-W scores = 142 and 104, respectively) than Orf22 is to Orf23 (N-W score = 31). ICE*Bs1* HelP is 126 amino acids, Tn*916* Orf22 is 128 amino acids, and Tn*916* Orf23 is 104 amino acids.

The well characterized ICE Tn*916* encodes two HelP homologues, Orf22 and Orf23. Comparison of HelP to each of these indicates that HelP is more similar to each protein than they are to each other (Figure 4). In addition, Orf23 is about 20 amino acids shorter at the C-terminus than Orf22 and HelP.

**Figure 5:**
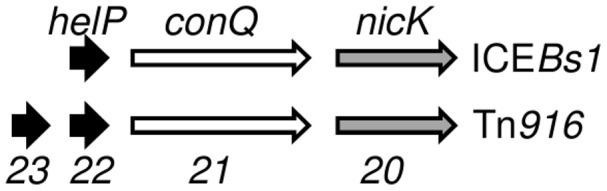
Organization of genes for HelP, ConQ (coupling protein) and NicK homologues in ICE*Bs1* and Tn*916*. Schematic diagram showing the organization of genes encoding HelP, ConQ and NicK in ICE*Bs1* and their homologues Orf23, Orf22, Orf21, and Orf20 in Tn*916*. Most (>40) of the 72 ICEs in the ICEberg database that have *helP* homologues have this consecutive (*helP*)-*helP*-*conQ*-*nicK* configuration. Some ICEs have the same gene order but have an additional one or two genes located between the *helP* and *conQ* and/or between *conQ* and *nicK*. Not shown is a different gene organization found in ICEs related to ICE*6013* in which the *conQ* is separate from *helP* and *nicK*.

The gene organization in ICE*Bs1* near *helP* and in Tn*916* near *orf22* and *orf23* is similar (Figure 5). *helP* and its homologues are usually grouped with two other genes: 1) a relaxase gene, *nicK* in ICE*Bs1* and *orf20* in Tn*916*, and 2) a gene encoding the predicted coupling protein, *conQ* in ICE*Bs1* [18] and *orf21* in Tn*916* (Figure 5). The coupling protein targets the relaxosome complex that is linked to ssDNA to the mating pore [28]. This genetic arrangement reflects a functional relationship as the relaxase and likely HelP are part of the relaxosome that interacts with the cognate coupling protein. When present, the majority of *helP* homologues are found in pairs, although ICE*Bs1* has only one *helP*.

**Figure 6:**
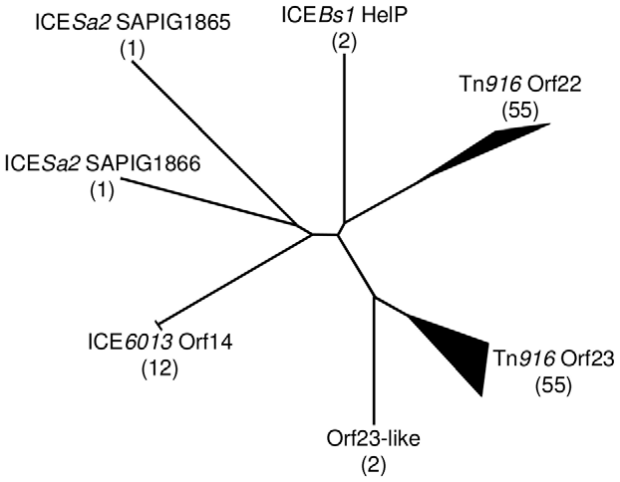
Phylogenic tree of HelP homologues. Using the ICEberg database and search tools (HMMER3 and WU-BLAST2) (http://db-mml.sjtu.edu.cn/ICEberg/), we identified 128 HelP homologues in 72 ICEs. The homologues were analyzed using CLUSTAL X and grouped into clades if they were within a distance of <0.22. The diagram is of an unrooted tree with the number of homologues in each clade shown in parentheses. Branch lengths indicate relative phylogenic distances. The width and length of branch tips indicate the number of homologues and the relative phylogenic distance between homologues in the clade, respectively. Of the seven clades, six are named for a representative ICE and its HelP homologue and one is named “Orf23-like” to reflect its close relationship to the Tn*916* Orf23 clade. Twelve homologues in the ICE*6013* Orf14 clade have the identical 106 amino acid sequence. The two homologues in the ICE*Bs1* HelP clade only differ by 5 of 126 amino acids. The two homologues in the Orf23-like clade only differ by 7 of 108 amino acids. Sixteen ICEs encode a single homologue of HelP. These sixteen homologues are from three clades - ICE*Bs1* HelP (2), ICE*6013* Orf14 (12) and Tn*916* Orf23 (2). The remaining 56 ICEs encode pairs of HelP homologues. The members of each pair of homologues fall into separate clades as exemplified by Tn*916* Orf22 (128 aa) and Tn*916* Orf23 (104 aa), and ICE*Sa2* SAPIG1865 (141 aa) and ICE*Sa2* SAPIG1866 (107 aa).

*helP* homologues are found in at least 72 ICEs or putative ICEs, primarily in firmicutes [29]. Phylogenic analysis revealed that these HelP homologues fall into seven clades (Figure 6). When an ICE encodes two HelP homologues, each one is in a separate clade: one clade contains the longer HelP homologue and other clade contains the shorter HelP homologue. For example the clade labeled “Tn*916* Orf22” contains most of the longer HelP homologues and the clade labeled “Tn*916* Orf23” contains most of the shorter HelP homologues. ICE*Bs1* HelP is similar in size to the longer Orf22, but appears to be almost equidistant from the Orf22 and Orf23 clades (Figure 6). Together, our results indicate that HelP proteins are encoded by many different ICEs from Firmicutes. If the function of these homologues is conserved, then HelP proteins act as helicase processivity factors for many ICEs and these ICEs likely undergo autonomous rolling circle replication.

## Discussion

**Figure 7:**
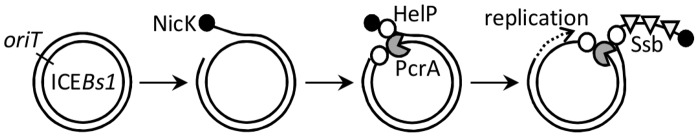
Model for association of the relaxase, helicase, HelP, and Ssb with ICE*Bs1* DNA. Following excision from the chromosome (not shown), the double-stranded circular ICE*Bs1* DNA is nicked at *oriT* by the relaxase NicK (filled circle). By analogy to related relaxases, NicK likely becomes covalently attached to the 5′ end of the nicked DNA on the strand that is transferred during conjugation. HelP (open circles) and the host-encoded helicase PcrA (gray packman) associate with the nicked ICE*Bs1* at *oriT*. HelP facilitates processive unwinding of ICE*Bs1* by PcrA and subsequent association of the host-encoded Ssb (open triangles). Unwinding of ICE*Bs1* by PcrA and HelP is required for replication and conjugation of ICEBs1. HelP is depicted as binding to both single strands of ICE*Bs1* DNA adjacent to PcrA, although it is possible that HelP associates only with one of the two single strands.

We found that the ICE*Bs1 helP* gene product stimulates unwinding of ICE*Bs1* DNA by the helicase PcrA in vivo. In vitro, HelP stimulated processivity of the PcrA-like helicase UvrD from *E. coli*. Our findings indicate that HelP is a helicase processivity factor that is required for conjugation and replication of ICE*Bs1*. HelP may represent a new family of helicase processivity factors as *helP* homologues are found in many other known and putative ICEs. Together, our results indicate that the order of events leading to ICE*Bs1* replication and conjugation include: 1) nicking by the ICE*Bs1*-encoded relaxase NicK; 2) association of HelP and the host encoded helicase PcrA, independently of each other, with the nicked ICE*Bs1* DNA template; and 3) unwinding of ICE*Bs1* dsDNA by PcrA and association of the host Ssb with the ICE*Bs1* ssDNA (Figure 7).

### DNA translocase and helicase activities of PcrA-family proteins

PcrA and its well-characterized homologues in *E. coli*, UvrD and Rep, are members of superfamily 1 (SF1) of non-hexameric helicases {reviewed in [13], [26]}. These proteins are highly processive 3′-5′ ssDNA translocases that bind to and move along strands of ssDNA. They are also weak 3′-5′ helicases that bind and destabilize dsDNA. They are involved in multiple cellular processes, including several types of DNA repair, replication restart, and clearing recombination proteins from DNA {reviewed in [26], [30]}. Strains lacking SF1 helicases are usually nonviable [24], [31], presumably due to loss of the DNA translocase activity of these proteins [31]–[33].

Although PcrA, Rep and UvrD are efficient and processive DNA translocases, they have very poor helicase activity on their own [19], [34], [35]. Helicase activity requires oligomerization [36]–[38], and these helicases interact with accessory factors that stimulate activity. For example, the DNA mismatch repair protein MutL facilitates loading and processivity of UvrD in *E. coli* [39] and the double-strand break repair protein Ku interacts with and stimulates processivity of UvrD1 in *Mycobacterium smegmatis* [40]. PcrA processivity is stimulated in vitro by YxaL from *B. subtilis* [41], although the role of YxaL in vivo is not known.

### Plasmid- and phage-encoded helicase processivity factors

Although they are poorly processive, PcrA, UvrD, and Rep are used by many plasmids and phages that undergo rolling-circle replication. In *E. coli*, UvrD is needed for rolling circle replication of some plasmids [27], [42] and Rep is used for replication of several ssDNA phages {reviewed in [11]}. In some Gram positive bacteria, PcrA is used for replication of many different rolling circle plasmids [12], [31], [32]. In addition, PcrA is required for unwinding of ICE*Bs1* DNA for both conjugation and replication [6].

For rolling circle replicating elements that use a host-encoded SF1-type helicase, efficient unwinding of duplex DNA is often stimulated by interaction with the element-encoded replication relaxase. Relaxases introduce a nick into dsDNA and mark the site for recruitment of the helicase for unwinding and replication of the plasmid or phage DNA. Replicative relaxases of ssDNA phages from *E. coli*, including gpA (cisA) from øX174 and the product of gene II of the f1 family of phages, increase Rep helicase processivity {reviewed in [11]}. The replicative relaxases of rolling circle replicating plasmids pT181 and the related pC221, RepC and RepD, respectively, appear to interact with and stimulate PcrA [23], [43]–[47]. In the case of RepD, this stimulation is thought to occur by increasing the affinity of PcrA for its DNA substrate and potentially by decreasing its rate of dissociation, rather than by altering its kinetic properties [46], [48].

### HelP, a conserved helicase processivity factor

As far as we are aware, all known examples of plasmid or phage-encoded helicase-stimulating proteins are relaxases. By analogy to the related plasmid relaxases, we expected that the only ICE*Bs1* product needed for ICE*Bs1* rolling circle replication would be its relaxase NicK. However, we found that the ICE*Bs1* gene product HelP is a helicase processivity factor that is required, in addition to NicK, for replication and conjugation of ICE*Bs1*. We do not yet know how HelP stimulates helicase activity, but it appears to act at a step after association of PcrA with ICE*Bs1 oriT* DNA. HelP could stimulate dimerization of PcrA since it is the dimer and not the monomer that has helicase activity [19]. HelP could also decrease the rate of dissociation of PcrA from DNA during unwinding, analogous to the activity demonstrated for RepD [46], [48]. HelP could accomplish one or a combination of these stimulatory functions by direct protein-protein contact with PcrA. Alternatively, HelP could remodel the DNA substrate during unwinding, promoting helicase activity.

*helP* homologues are found primarily in firmicutes. There appear to be >300 homologues in the non-redundant protein database, and at least 128 of these are found in known or putative ICEs that are included in the ICEberg database [29], and we suspect that most of the others are in unrecognized ICEs. Although ICE*Bs1* has only one *helP*, most, but not all, *helP* homologues are found in pairs, with one member of the pair approximately 20 amino acids longer than the other. It is possible that each member of a HelP pair stimulates processivity of a dissimilar range of helicases, thereby broadening the host range of the mobile element. If the function of HelP homologues is conserved, then the ICEs with *helP* homologues likely undergo autonomous replication and that HelP proteins are important for stability and transfer of many ICEs.

### Other strategies for DNA unwinding of extrachromosomal elements

In contrast to the many ICEs and plasmids that utilize a host-encoded SF1-type helicase for replication and transfer and rely on an accessory factor to facilitate unwinding, some ICEs and plasmids encode their own SF1-type helicase. PcrA-like helicase domains are found attached to some plasmid relaxases. For example, TraI of the *E. coli* F plasmid and TrwC of R388, have a C-terminal PcrA-like helicase domain that is required for conjugation [49]–[51]. The helicase activity is highly processive, potentially because it is tethered to the relaxosome {reviewed in [52]}. In addition, many known and putative ICEs encode discrete PcrA-like helicases [29]. It is not known if they are processive or require accessory proteins, although some of those ICEs also contain a *helP* homologue. An element that encodes its own helicase might have a broader host range by avoiding reliance on a host-encoded helicase. That HelP homologues, relaxases that also function as processivity factors, and PcrA homologues are encoded on conjugative elements indicates that the need for DNA unwinding can be met in different ways.

## Materials and Methods

### Strains and alleles

All *B. subtilis* strains (Table 4) are derivatives of JH642 (*trpC2 pheA1*) and were constructed by natural transformation. Strains are either cured of ICE*Bs1* (designated ICE*Bs1* ^0^) or contain ICE*Bs1* marked with the Δ(*rapI*-*phrI*)*342*::*kan* allele to monitor conjugative transfer [9]. ICE*Bs1* gene expression was induced by overproduction of *rapI* from *amyE*::{(Pspank(hy)-*rapI*) *spc*}, *amyE*::{(Pxyl-*rapI*) *spc*}, or *lacA*::{(Pxyl-*rapI*) *tet*}. Strains used as recipients in mating experiments contained the streptomycin resistance allele (*str-84*) [9]. Other mutations that were previously described include: the deletion-insertion Δ*pcrA1021*::*cat* [6]; *thrC1165*::*cat* [6], used as a control for threonine auxotrophy and chloramphenicol resistance; and *thrC329*::{(Pxis-*nicK*) *mls*} [15], used for ectopic expression of *nicK*.

**Table 4:** Strains and plasmids.

Strain	Relevant Genotype
CAL85	ICE*Bs1* ^0^ *str*-*84*
CAL306	Δ*nicK306* Δ(*rapI*-*phrI*)*342*::*kan amyE*::{(Pspank(hy)-*rapI*) *spc*}
CAL333	*thrC329*::{(Pxis-*nicK*) *mls*}
CAL346	Δ*nicK306* Δ(*rapI*-*phrI*)*342*::*kan amyE*::{(Pspank(hy)-*rapI*) *spc*} *thrC329*::{(Pxis-*nicK*) *mls*}
CAL1131	Δ(*rapI-phrI*)*342*::*kan lacA*::{(Pxyl-*rapI*) *tet*} Δ*recF*::*spc*
CAL1144	Δ(*rapI-phrI*)*342*::*kan lacA*::{(Pxyl-*rapI*) *tet*} Δ*recF*::*spc* Δ*pcrA1021*::*cat*
CAL1686	Δ(*rapI-phrI*)*342*::*kan amyE*::{(Pxyl-*rapI*) *spc*} Δ*pcrA1021*::*cat thrC1642*::{(Pspank*-uvrD*) *mls*}
CAL1772	Δ(*rapI-phrI*)*342*::*kan amyE*::{(Pxyl-*rapI*) *spc*} *thrC1165*::*cat*
CAL1773	Δ(*rapI-phrI*)*342*::*kan amyE*::{(Pxyl-*rapI*) *spc*} *thrC1642*::{(Pspank*-uvrD*) *mls*}
IRN342	Δ(*rapI*-*phrI*)*342*::*kan*
JMA168	Δ(*rapI*-*phrI*)*342*::*kan amyE*::{(Pspank(hy)-*rapI*) *spc*}
JMA222	ICE*Bs1* ^0^
JT148	ICE*Bs1* ^0^ *thrC148*::{(Pspank(hy)-*helP*) *mls*}
JT155	Δ*helP155* Δ(*rapI*-*phrI*)*342*::*kan amyE*::{(Pspank(hy)-*rapI*) *spc*}
JT162	Δ(*rapI-phrI*)*342*::*kan* ICE*Bs1*::pCAL1255 (*oriT*, Pxis*-helP-nicK*)
JT163	Δ(*rapI-phrI*)*342*::*kan* ICE*Bs1*::pCAL1260 (*oriT*, Pxis*-helP*)
JT164	Δ(*rapI-phrI*)*342*::*kan* ICE*Bs1*::pJT151 (*oriT*, Pxis*-nicK*)
JT165	ICE*Bs1* ^0^ pCAL1255 (*oriT*, Pxis*-helP-nicK*)
JT167	ICE*Bs1* ^0^ *thrC148*::{(Pspank(hy)-*helP*) *mls*} pJT151 (*oriT*, Pxis*-nicK*)
JT216	ICE*Bs1* ^0^ *thrC148*::{(Pspank(hy)-*helP*) *mls*} *str*-*84*
JT252	Δ*helP155* Δ(*rapI-phrI*)*342*::*kan amyE*::{(Pspank(hy)-*rapI*) *spc*} *lacA*::{(*rpsF-ssb-mgfpmut2*) *tet*]
JT308	Δ(*rapI*-*phrI*)*342*::*kan nicK-myc amyE*::{(Pspank(hy)-*rapI*) *spc*}
JT334	Δ*helP155 thrC229*::{(ICE*Bs1*-*1635* Δ*helP*-*attR*::*cat*) *mls*} Δ(*rapI*-*phrI*)*342*::*kan amyE*::{(Pspank(hy)-*rapI*) *spc*}
JT335	Δ*helP155 thrC229*::{(ICE*Bs1*-*1637* Δ*conQ*-*attR*::*cat*) *mls*} Δ(*rapI*-*phrI*)*342*::*kan amyE*::{(Pspank(hy)-*rapI*) *spc*}
JT340	Δ(*rapI*-*phrI*)*342*::*kan nicKY195F-myc amyE*::{(Pspank(hy)-*rapI*) *spc*}
JT397	Δ*helP155* Δ(*rapI-phrI*)*342*::*kan amyE*::{(Pspank(hy)-*rapI*) *spc*} *lacA*::{(*rpsF-ssb-mgfpmut2*) *tet*] *thrC229*::{ICE*Bs1*-*1635* Δ*helP*-*attR*::*cat*) *mls*}
JT398	Δ*helP155* Δ(*rapI-phrI*)*342*::*kan amyE*::{(Pspank(hy)-*rapI*) *spc*} *lacA*::{(*rpsF-ssb-mgfpmut2*) *tet*} *thrC229*::{ICE*Bs1*-*1637* Δ*conQ*-*attR*::*cat*) *mls*}
JT403	ICE*Bs1* ^0^ *thrC329*::{(Pxis-*nicK*) *mls*} pCAL1260 (*oriT*, Pxis*-helP*)
MMB834	Δ(*rapI-phrI*)*342*::*kan amyE*::{(Pspank(hy)-*rapI*) *spc*} *lacA*::{(*rpsF-ssb-mgfpmut2*) *tet*}

#### *helP*

*ΔhelP155* is an unmarked 413-bp deletion that removes the entire coding sequence and the 35 bp *helP-ydcQ* intergenic region (Figure 1), constructed using the same method as for the Δ*nicK306* allele [15]. Complementation of Δ*helP155* was tested using a truncated ICE*Bs1* derivative integrated at *thrC*, *thrC229*::{(ICE*Bs1*-*1637* Δ*conQ*-*attR*::*cat*) *mls*} that is missing all of the ICE*Bs1* genes downstream from *helP* (Figure 1D), and contains *helP* and all the upstream ICE*Bs1* genes [18]. The control that does not complement *ΔhelP* was essentially the same ICE*Bs1* insertion at *thrC*, but with a deletion that removes *helP* (Figure 1C), *thrC229*::{(ICE*Bs1*-*1635* Δ*helP*-*attR*::*cat*) *mls*}.

Expression of *helP* from LacI-repressible-IPTG-inducible promoter Pspank(hy) [53] in *B. subtilis* was from a fusion at *thrC*, *thrC148*::{(Pspank(hy)-*helP*) *mls*}. This was made by amplifying *helP* by PCR from *B. subtilis* genomic DNA, digesting with Nhe1 and ligating into Nhe1-cut pCAL215 [16] to give pCAL890. The Pspank promoter of pCAL890 was altered to Pspank(hy) by Quikchange mutagenesis to give the plasmid pJT146 which was then used to integrate Pspank(hy)-*helP mls* into the *thrC* locus by double-crossover recombination.

#### *uvrD*

*E. coli uvrD* was expressed in *B. subtilis* from Pspank inserted at *thrC*, *thrC1642*::{(Pspank*-uvrD*) *mls*}. *uvrD* was amplified from an *E. coli* MC1061-derived strain and then inserted between the HindIII and SphI sites of pCAL215 [16] using isothermal assembly [54].

*pcrA* is essential in *B. subtilis* [24], [31]. The presence of *thrC1642*::{(Pspank*-uvrD*) *mls*} suppressed the lethality caused by loss of *pcrA*. That is, Δ*pcrA*::*cat* could be transformed into competent cells containing the Pspank-*uvrD*, but could not be transformed into cells without *uvrD*. Growth of the Pspank-*uvrD* Δ*pcrA* mutant was independent of IPTG, indicating that the leaky (uninduced) expression from Pspank-*uvrD* was sufficient to compensate for the absence of *pcrA*.

#### *nicK*

The *nicK-myc* allele was constructed by allelic replacement using essentially the same method as for the Δ*helP155* allele. An approximately 2.1 kb DNA insert containing *nicK*, a C-terminal 3× *c-myc* tag and 897 bp of ICE*Bs1* sequence downstream of *nicK* were cloned into the plasmid pCAL1422 by isothermal assembly [54] to give the plasmid pJT296. pCAL1422 contains *cat* (chloramphenicol-resistance in *B. subtilis*) and *E. coli lacZ* under control of the constitutive promoter Ppen [55]. The tyrosine codon at position 195 of *nicK-myc* in pJT296 was changed to a phenylalanine codon, *nicKY195F-myc*, by quick-change mutagenesis (Stratagene), giving plasmid pJT318. pJT296 and pJT318 were used to replace *nicK* on ICE*Bs1* with *nicK-myc* and *nicKY195F-myc*, respectively, by first integrating by single crossover and then screening for loss of the plasmid by virtue of loss of *lacZ*, and then testing by PCR for introduction of the indicated allele, essentially as described [15].

Wild type NicK fused to the Myc-tag (NicK-Myc) was active as judged by normal conjugation efficiencies (approximately 1.2% transconjugants per donor). In contrast, the *nicKY195F-myc* mutant was defective in conjugation (<0.00008% transconjugants per donor).

### Plasmids

Plasmids pCAL1255 (*oriT*, Pxis-*helP-nicK*), pCAL1260 (*oriT*, Pxis-*helP*), and pJT151 (*oriT*, Pxis-*nicK*) contain DNA segments from ICE*Bs1* inserted into the BamHI restriction site of pUS19, a spectinomycin-resistant derivative of pUC19 [14]. In addition to conferring resistance to spectinomycin in *B. subtilis*, each plasmid contains ICE*Bs1 oriT*.

pCAL1255 (*oriT*, Pxis-*helP-nicK*) contains the *xis* promoter (Pxis) driving transcription of *helP* and *nicK*. The P*xis-helP-nicK* insertion is comprised of three non-contiguous segments of ICE*Bs1*: 1) a 527 bp segment containing 254 bp of the 5′-end of *immR* and the entire 273 bp intergenic region between *immR* and *xis*; 2) a 419 bp segment that contains *helP* and extends 35 bp downstream of the *helP* stop codon; and 3) a 1076 bp segment containing *nicK* (and *oriT*) and extending 17 bp downstream of the *nicK* stop codon. The junctions between the DNA segments were designed to allow translation initiation of *helP* from the *xis* ribosome binding site and translation initiation of *nicK* from the *conQ* ribosome binding site, as the start codons of *helP* and of *nicK* were placed the same distance downstream of the Pxis and the *helP*-*conQ* intergenic regions as the native start codons of *xis* and *conQ*, respectively. Transcription of the P*xis*-*helP*-*nicK* insertion is in the same orientation as the spectinomycin- and ampicillin-resistance genes on the vector backbone.

pCAL1260 (*oriT*, Pxis-*helP*) contains the same P*xis*-*helP*-*nicK* insertion present in pCAL1255, but with an additional T inserted between the 5^th^ and 6^th^ codon of *nicK*. The single base insertion in pCAL1260 leads to premature termination after the 10^th^ codon of the *nicK* ORF. pJT151 is essentially pCAL1255 (*oriT*, Pxis-*nicK*) but with the entire *helP* coding sequence deleted.

N-terminal hexahistidine tagged HelP (his-HelP) and UvrD (his-UvrD) were overproduced in and purified from *E. coli*. *helP* was amplified by PCR from *B. subtilis* (strain JH642) chromosomal DNA, digested with BamHI and HindIII and ligated into the same sites of the T7-expression vector pET28b (Novagen) to give plasmid pCAL1297. *uvrD* was amplified from *E. coli* strain MC1061and cloned, by isothermal assembly, into the XbaI and BamHI sites of pET28b to give plasmid pJT370.

### Conjugation experiments

For conjugation experiments, all donor strains contained ICE*Bs1* with the Δ(*rapI*-*phrI*)*342*::*kan* allele and ICE*Bs1* gene expression was induced by overproduction of the regulatory protein RapI from an ectopic locus [9]. When donors carried the Pspank(hy)-*rapI* allele, all strains were grown in rich medium and induction was for one hour with 1 mM IPTG. When the Pxyl-*rapI* was used, strains were grown in minimal S750 medium containing 1% arabinose and induction was for 2 hours after the addition of 1% xylose [56]. Recipients were streptomycin resistant (*str-84*). Donors and recipients were mixed in a 1∶1 ratio, filtered onto nitrocellulose membranes and incubated on agar containing minimal salts for 3 hours, essentially as described [9]. The mating efficiency was determined as the number of colony forming units (CFU) of streptomycin- and kanamycin- resistant transconjugants per CFU of the donor.

### Chromatin immunoprecipitation, copy number, and qPCR

Association of HelP, PcrA, NicK-Myc and Ssb-GFP with ICE*Bs1* DNA was measured by chromatin immunoprecipitation followed by quantitative PCR (ChIP-qPCR) essentially as described [6], [57]. Polyclonal antibodies from rabbits (Covance) were used to precipitate HelP, PcrA and Ssb-GFP and monoclonal antibodies to c-Myc (Invitrogen) were used to precipitate NicK-Myc.

qPCR was used to measure the relative amount of DNA in immunoprecipitates and to measure relative plasmid copy number. Primers to the ICE*Bs1 nicK*/*oriT* region, designated *oriT*, were CLO280, 5′-TGGCTACGTT GGCACGTATG-3′, and CLO281, 5′-AATTGACGGC AACCTTGACC-3′. Primers to ICE*Bs1 conE*, approximately 6 kb downstream from *oriT*, were oMMB238, 5′-TGATGGTTCAAATCCTGCATTGTCAC-3′, and oMMB239, 5′-GAACTTACCT AGTGCAAACATGAC-3′.

Plasmid copy number was determined using primers oJT168, 5′-GTGGAATCAT CCTCCCAAAC-3′, and oJT169, 5′-AATGGCTCTT CTCACATCAG-3′, that are specific to *spcE* found on the plasmids and not in the chromosome.

Values obtained in ChIP-PCR and plasmid copy number experiments were normalized to the chromosomal locus *ydbT*, approximately 15 kb upstream of the chromosomal attachment site for ICE*Bs1*, with primers CLO284, 5′-CTTCCGCACA TGCTCCGAAC-3′ and CLO285, 5′-TCGGCAGCAG GATCACTGAC-3′.

### Protein expression and purification

his-HelP and his-UvrD were purified using similar conditions. Expression strains were grown in 2 liters of 2×YT medium supplemented with 0.4% glycerol, 20 mM sodium phosphate buffer pH 7.0, and 10 mM MgSO_4_ at 30°C until they reached an OD600 of 0.8. Protein expression was induced by the addition of 0.2% arabinose and 1 mM IPTG followed by incubation for 3 hours. Cells were harvested by centrifugation, washed with 100 ml of phosphate-buffered saline, resuspended in 25 ml lysis buffer {50 mM Tris-HCl ph 8.0, 0.3 M NaCl, 10 mM imidazole, 1 mg/ml lysozyme, 5 µg/ml DNase I and 1× CellLytic Express (Sigma-Aldrich)} and lysed by incubation at room temperature with gentle inversions. The lysate was cleared by centrifugation at 15,000× g and the cleared lysate was applied to 1 ml Ni-NTA agarose resin, washed with Tris-NaCl (50 mM Tris-HCl pH 8.0, 0.3 M NaCl) containing 10 and 20 mM imidazole and eluted with the same buffer containing 250 mM imidazole. The eluted protein was dialyzed against 10 mM MOPS, pH 7.5, 200 mM NaCl and 1 mM tris(2-carboxyethyl)phosphine. his-UvrD was approximately 93% pure and his-HelP was approximately 97% pure.

### Helicase assays

Helicase activity was measured using two different partial-duplex DNA substrates, designed by analogy to previously described templates [41]. The substrates were generated by annealing M13mp18 circular ssDNA (Affymetrix) with 1) a 22-bp oligonucleotide (oJT276, 5′-ACTCTAGAGGA TCCCCGGGTAC-3′), or 2) an 81-bp oligonucleotide (oJT278, 5′-GGCCAGTGCCA AGCTTGCATG CCTGCAGGTC GACTCTAGAG GATCCCCGGG TACCGAGCTC GAATTCGTAA TCATGGTCAT-3′). Each oligonucleotide was labeled on the 5′-end with an infrared fluorescent dye, IRDye800 for oJT276 and TYE705 for oJT278 (IDT).

Helicase assays were performed in 200 µl reactions at 37°C. Mixtures containing 0.5 nM DNA substrate and 0 or 1 µM his-HelP, in 20 mM MOPS, pH 7.5, 200 mM NaCl, 15 mM MgCl2, 2.5 mM ATP, 1 mM tris(2-carboxyethyl)phosphine were preincubated at 37°C for 10 min, and started by the addition of 25 nM his-UvrD. 20 µl aliquots were withdrawn at regular intervals and added to 5 µl stop buffer (5% Ficoll, 15% glycerol, 0.12% Orange G, 1% SDS and 50 mM sodium EDTA). Samples (10 µl) were analyzed by electrophoresis on a 15% TBE-polyacrylamide gel containing 2.5% glycerol followed by visualization and quantitation using the Odyssey Infrared imaging system (Li-Cor).

### Identification and analysis of HelP homologues

We identified 128 homologues of ICE*Bs1 helP* (including *helP*) in 72 bacterial ICEs using the HMMER3 and WU-BLAST2 search tools in ICEberg (http://db-mml.sjtu.edu.cn/ICEberg/), the web-based resource for bacterial ICEs . The ICEberg database (last updated on August 14, 2011) contains sequence information for 431 known and putative ICEs. All 130 homologues were aligned using ClustalX 2.1 [58] using multiple-alignment mode with default parameters. The neighbor-joining method was used to generate the phylogenic tree from the ClustalX PHYLIP output file at the Interactive Tree of Life (http://itol.embl.de/index.shtml) [59]. Seven clades were defined by grouping together homologues that had an average distance of <0.22.

An additional HMMER search [60] with default search settings (hmmer.janelia.org) identified >300 HelP homologues in the non-redundant protein database. All but five of these were in Firmicutes. The exceptions were one homologue in a Mycoplasma, Ureaplasma urealyticum, two in an uncultured bacterium MID12, and two homologues in *Klebsiella pneumoniae* which carries Tn*916* (usually associated with Firmicutes) on the composite ICE Tn*6009*.

Global sequence similarities between HelP and the two HelP homologues from Tn*916* were analyzed by the Needleman-Wunsch alignment method [61].
